# Effect of a Combination of Myo-Inositol, Alpha-Lipoic Acid, and Folic Acid on Oocyte Morphology and Embryo Morphokinetics in non-PCOS Overweight/Obese Patients Undergoing IVF: A Pilot, Prospective, Randomized Study

**DOI:** 10.3390/jcm9092949

**Published:** 2020-09-12

**Authors:** Stefano Canosa, Carlotta Paschero, Andrea Carosso, Sara Leoncini, Noemi Mercaldo, Gianluca Gennarelli, Chiara Benedetto, Alberto Revelli

**Affiliations:** Gynecology and Obstetrics 1, Physiopathology of Reproduction and IVF Unit, S. Anna Hospital, Department of Surgical Sciences, University of Torino, Via Ventimiglia 3, 10126 Torino, Italy; carlotta.paschero@libero.it (C.P.); andrea88.carosso@gmail.com (A.C.); rasi89@hotmail.it (S.L.); noemimercaldo@gmail.com (N.M.); gennarelligl@gmail.com (G.G.); chiara.benedetto@unito.it (C.B.); aerre99@yahoo.com (A.R.)

**Keywords:** Myo-Inositol, oocyte quality, polarized light microscopy, embryo morphokinetics, overweight patients, obesity, IVF

## Abstract

Herein we aimed at assessing whether Myo-Inositol (MI), Alpha–Lipoic acid (ALA), and Folic acid (FA) could improve oocyte quality and embryo development in non-PCOS overweight/obese women undergoing IVF. Three hundred and twenty-four mature oocytes were obtained from non-PCOS overweight/obese patients, randomized to receive either MI, ALA, and FA (MI + ALA + FA group, *n* = 155 oocytes) or FA alone (FA-only group, *n* = 169 oocytes). Oocytes were examined using Polarized Light Microscopy to assess morphological features of zona pellucida (ZP) and meiotic spindle (MS). One hundred and seventy-six embryos (*n* = 84 in the MI + ALA + FA group, *n* = 92 in the FA-only group) were assessed by conventional morphology on days 2 and 5, as well as using the Time-Lapse System morphokinetic analysis. A significantly higher ZP retardance, area, and thickness (*p* < 0.05), and a shorter MS axis (*p* < 0.05) were observed in the MI + ALA + FA group, suggesting a positive effect on oocyte quality. Conventional morphology evaluation on day 2 showed a higher mean embryo score in the MI + ALA + FA group, whereas embryo morphokinetic was comparable in the two groups. Overall, our data show a possible beneficial effect of the combination of MI, ALA, and FA on oocyte and embryo morphology, encouraging testing of this combination in adequately powered randomized trials to assess their impact of clinical IVF results.

## 1. Introduction

Oocyte quality is a pivotal factor in determining IVF outcome, and the interest in the follicular microenvironment in which oocytes develop is witnessed by a series of recent studies [1,2,3]. Supplementation with antioxidants, vitamins, or hormones before and during controlled ovarian stimulation (COS) has been proposed as an attempt to improve the follicular microenvironment and, in turn, oocyte quality [4,5,6]. Myo-Inositol (MI), a natural compound with insulin-sensitizing properties, has been associated with improved ovarian function in both women with polycystic ovary syndrome (PCOS) [7,8] and poor responders [9]. Indeed, MI might act through other mechanisms than insulin sensitization, e.g., by promoting the nuclear maturation of germinal vesicle oocytes [10]. In humans, treatment with MI combined with folic acid (FA) reduced the proportion of immature and degenerated oocytes, thus increasing the total amount of mature oocytes [11]. 

As for FA, a large amount of literature regarding its administration during the peri-conceptional period confirms a positive effect in preventing fetal neural tube defects [12], and the WHO recommends its use until 12 weeks of gestation [13]. In women undergoing IVF, FA intake increases follicular fluid folate concentration, decreasing homocysteine concentration and limiting its detrimental effects on oocyte quality [14]. In addition to MI and FA, other molecules with antioxidant properties could improve oocyte competence: Alpha-Lipoic acid (ALA), which acts as a cofactor for pyruvate dehydrogenase, and Alpha-ketoglutarate dehydrogenase in mitochondria, which exerts a positive impact on the development of mouse pre-antral follicles [15]. More recently, the combination of three antioxidants (acetyl-L-carnitine, N-acetyl-L-cysteine, and ALA) has been shown to improve embryonic and foetal development in a murine IVF model, suggesting a positive effect on oocytes [16]. The combined treatment with MI, ALA, and FA has already been reported to improve sperm concentration, motility, and morphology in subfertile men [17]. However, no studies so far investigated the effects of this combination on human oocytes and on human embryo development in non-PCOS overweight/obese women. It was previously reported that some birefringent characteristics of the meiotic spindle (MS) and of the zona pellucida (ZP) are associated with a high level of structural organization of the human oocyte and positively related to the developmental potential of the deriving embryo [18,19]. On the other hand, morphokinetic features of the cleavage stage embryo were claimed to predict blastocyst development in vitro [20,21]. The present study was designed as a pilot randomized prospective study aimed at clarifying whether this combination of nutraceuticals, given during three months preceding IVF, could affect measurable oocyte quality parameters assessed by Polarized Light microscopy (PLM) and/or embryo morphology and in vitro development kinetics evaluated by Time-Lapse system (TLS) analysis. 

## 2. Materials and Methods

### 2.1. Patients

The study was designed as a pilot, prospective randomized trial. Aiming at evaluating approximately 350 mature oocytes and the embryos derived from them, we included in the study a total number of 40 women (mean age: 36.1 ± 4.6, range 25–40) with ovarian reserve markers (antral follicle count, AFC ≥ 8, and anti-mullerian hormone, AMH > 2.5 ng/mL), suggesting a normal responsiveness to gonadotropin stimulation, as previously described [22]. Patients were chosen among non-PCOS overweight/obese women (body mass index ≥ 25) undergoing intracytoplasmic sperm injection (ICSI) with own oocytes at our IVF Unit between February 2018 and September 2019. Patients with PCOS, diabetes mellitus (type I or II) or on anti-diabetic therapy, affected by autoimmune diseases, and/or with unfavorable markers of ovarian reserve (AMH < 0.5 ng/mL) were excluded from recruitment [23]. Patients were randomized (1:1) using a specific software available online (http://www.randomizer.org) and divided into two sub-groups: (a) patients taking 2 g MI + 800 mg ALA + 400 mg FA (Sinopol^®^, Laborest, Milan, Italy) daily for 3 months before COS (MI + ALA + FA group, *n* = 20), and (b) patients taking 400 mg FA only (Folidex, Italfarmaco Spa, Milan, It aly) daily for 3 months before COS (FA-only group, *n* = 20). The study was carried out in accordance to the Declaration of Helsinki and was authorized as a prospective study by the local Ethical Committee (Ref. number 005418). Signed, written informed consent was obtained from all patients.

### 2.2. Controlled Ovarian Stimulation (COS) and Oocyte Retrieval

Patients in both groups received a starting dose of 150–300 IU/day recombinant FSH (rFSH; Gonal F^®^; Merck, Germany), that was chosen according to their age, AFC and AMH. Circulating E2 and transvaginal US examination were performed every second day from stimulation day 6–7 to monitor follicular growth, adapting the FSH dose when required. When at least two follicles reached 18 mm mean diameter, with appropriate E2 levels, a single s.c. injection of 10,000 IU hCG (Gonasi HP, IBSA, Switzerland) was administered in order to trigger ovulation. US-guided oocyte pick-up (OPU) was performed 35–37 h later under local anesthesia (paracervical block). Follicular fluids were aspirated and immediately observed under a stereomicroscope. Cumulus-oocyte complexes (COCs) were washed in buffered medium (Flushing medium, Cook Ltd., Ireland) and after 2 h from OPU, oocytes and cumulus cells (CCs) were separated by gently pipetting in 40 μL HEPES-buffered medium containing 80 IU/mL hyaluronidase (ICSI Cumulase, Origio Medicult, Denmark); nuclearly mature, metaphase II (MII) oocytes were then examined using Polarized Light Microscopy (PLM) just before being injected.

### 2.3. Polarized Light Microscopy (PLM)

During PLM assessment, each MII oocyte was placed on a glass bottom dish (Willco Wells, Amsterdam, The Netherlands) in a 10 μL drop of buffered, pre-warmed medium, covered by mineral oil (Culture Oil, Cook Ltd., Ireland), and was kept on a 37 °C stage warmer under the microscope (CRi Oosight™, Woburn, MA, USA). PLM images of each oocyte were collected at 400× magnification and recorded. The Oosight Meta™ software, allowing automatic zona pellucida (ZP) and meiotic spindle (MS) detection, was used to acquire and analyze data as previously described [18]. The following parameters were automatically measured: average retardance, area and thickness of the inner layer (IL) of the ZP, average retardance and area of the MS. In addition, the major axis of the MS was manually measured using a line scan.

### 2.4. Preparation of Semen Samples and Intra-Cytoplasmatic Sperm Injection (ICSI)

Semen samples were examined to assess sperm concentration, motility, and morphology according to the World Health Organization guidelines (WHO laboratory manual, 2010), and were prepared by density gradient centrifugation in order to select normally motile and morphologically normal spermatozoa. PLM assessment required CCs removal so that all the enrolled patients underwent sperm injection, irrespective of the quality of semen samples. ICSI was performed on all available MII oocytes approximately 2 h after CCs removal, and injected oocytes were then placed in culture dishes for further morphokinetic analysis.

### 2.5. Fertilization Check, Embryo TLS Analysis and Embryo Transfer

Normal fertilization was assessed by evaluating the presence of two pronuclei (2PN) and the extrusion of the second polar body after 16–18 h from sperm injection. Normally fertilized oocytes (zygotes) were further cultivated in the Time-Lapse System Geri plus^®^ (Genea Biomed, Merck, Germany) at 37 °C in humidified atmosphere (5% O_2_, 6% CO_2_ balanced with N_2_), in micro-wells with integrated monitoring system (16 wells/dish; one zygote/well). The shape of this dish allows following each embryo individually even if all embryos of a given patient share a common 80 μL drop of medium. Embryos were cultured in pre-equilibrated Cleavage medium (Cook, Ireland) overlaid with mineral oil up to day 3 of development; at this stage, a change of medium was performed using a stage-specific medium (Blastocyst medium, Cook, Ireland) until the blastocyst stage. As usual practice in our IVF Lab, embryo morphological assessment was performed evaluating embryo morphology on day 2 using the Integrated Morphology Cleavage Score (IMCS) [24], and on day 5 according to The Istanbul Consensus Workshop [25]. IMCS is a score constructed to be evidence-based, as it was obtained comparing implanted embryos vs. non-implanted embryos in a rather large number of IVF cycles ending in double embryo transfer (DET) [24]. It is a ten-point scale score where the top quality embryos are those with score ≥ 8. In addition, bright field multiple images (11 different focal planes for each embryo) were captured by the Geri plus^®^ TLS every 5 min from the zygote stage to the blastocyst stage. Videos were then analyzed and the following morphokinetic parameters (times) were considered according to the ESHRE good practice recommendations [26]: pronuclear appearance (tPNa), pronuclear fading (tPNf), completion of cleavage to two, three, four and eight cells (t2, t3, t4, and t8 respectively), time of initiation of blastulation (tSB), time of full blastocyst development (tB), time of herniation or initiation of hatching process (tHN). The following time intervals were also calculated: tPNf–tPNa, t2–tPNf, t3–t2, t4–t3, t4–t2 and t8–t4. Finally, the optimal cleavage ranges were assessed in each group based on previously observations obtained comparing implanted versus not implanted embryos (t2: 24.3–27.9 h; t3: 35.4–40.3 h; t4: 36.4–41.6 h; t3–t2: ≤ 11.9 h; t4–t3: ≤ 0.76 h) [27]. All embryos showing events of direct or reverse cleavage and internalization of cellular fragments (≥ 30% of total volume) at TLS analysis were excluded from embryo transfer (ET). In order to ensure consistency in the evaluation, the morphological score and the morphokinetic analysis of all embryos were performed by the same embryologist in blind (he was not aware of the group to which the patient belonged). A total number of 40 ETs were performed, transferring either two embryos at the cleavage stage or a single blastocyst on day 5; the choice was driven by the number of fertilized oocytes, of top quality embryos on day 2, and of previous IVF attempts. The proportion of ETs performed at the blastocyst stage was 30% in the MI + ALA + FA group, and 50% in the FA-only group. ET was performed using the soft catheter Sydney Guardia (Cook, Australia) under trans-vaginal ultrasound guidance, applying the technique that was previously published by our group [28]. 

### 2.6. Statistical Analysis

The aim of this pilot study was to evaluate whether the administration of MI, ALA and FA before IVF could affect oocyte morphology and embryo kinetics. Secondarily, other IVF related outcomes were registered: implantation rate (IR), clinical (US confirmed) pregnancy rate (CPR), ongoing pregnancy rate at ≥ 20 weeks of gestation (OPR) and live birth rate (LBR). Continuous variables are shown as mean ± standard deviation (SD), whereas categorical variables are expressed as absolute values and percentage. After assessing the normal distribution of data by the Shapiro-Wilk test, comparison among groups was performed by GraphPad Prism V7 using the non-parametric Mann-Whitney test or the Chi-squared test, as appropriate. All statistical tests were two-sided and a *p* value of 0.05 or less was considered statistically significant.

## 3. Results

### Patients

**Figure 1:**
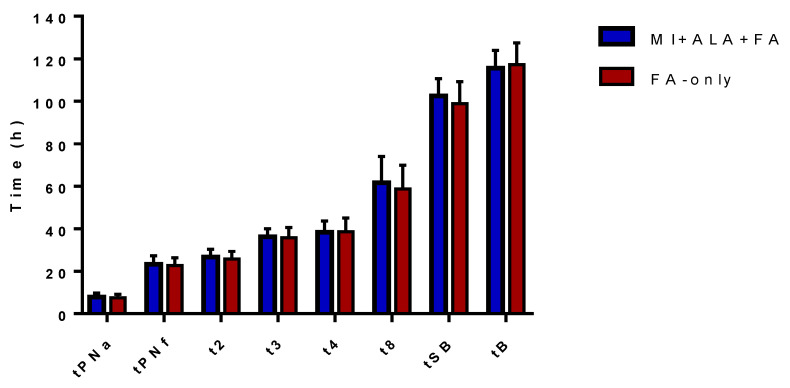
Morphokinetic parameters recorded by TLS GERI plus^®^. Times to the blastocyst stage are shown for embryos of the MI + ALA + FA group (patients assuming Myo-Inositol + Alpha-Lipoic acid + Folic acid) and of the FA-only group (patients assuming Folic acid alone). Absolute values are shown in Table 3 and expressed as mean ± standard deviation.

The clinical characteristics of the 40 enrolled patients, as well as the outcome of their IVF cycles, are summarized in Table 1; no significant differences were observed for age, BMI, AMH, and AFC. The slight difference in the mean age was not clinically relevant, as suggested by the comparable values of ovarian reserve biomarkers and by the mean number of retrieved oocytes. A significantly larger endometrial thickness at OPU was observed in the MI +ALA + FA group (11.4 ± 2.2 mm vs. 9.9 ± 1.8 mm; *p* < 0.05). A total number of 324 MII oocytes were collected and observed under PLM: a significantly higher inner layer retardance (*p* < 0.0001), larger area (*p* < 0.00001), and thickness (*p* < 0.05) of the ZP, and a significantly shorter MS axis (*p* < 0.05) were observed in the MI + ALA + FA group (*n* = 155 oocytes) compared to the FA-only group (*n* = 169 oocytes) (Table 2). Overall, 176 oocytes (84 in the MI + ALA + FA group and 92 in FA-only group) were normally fertilized. Embryo assessment by conventional morphology (IMCS) showed a significantly higher score (7.4 ± 2.3 vs. 6.7 ± 2.1; *p* < 0.01) and a higher proportion of top-quality embryos (45.2% vs. 26.1; *p* < 0.01) in the MI + ALA + FA group compared to FA-only group (Table 1). Embryo growth was further analysed using TLS Geri plus^®^: a comparable proportion of embryos undergoing direct cleavage to the three-cell stage (t3–t2 < 5 h) was observed (17.8% and 10.8% in the MI + ALA + FA and FA-only groups, respectively; Table 3 and Figure 1). More embryos displaying an optimal cleavage range for t2 (51.1% vs. 33.6%; *p* < 0.05) and t3 (47.6% vs. 25%; *p* < 0.01) were observed in the MI + ALA + FA group (Table 3). A total number of 58 fresh embryos (30 in the MI + ALA + FA group, 28 in FA-only group) were transferred in uteri: the implantation rate (IR) was 33.3% in the MI + ALA + FA group vs. 10.7% in FA-only group (*p* < 0.05) (Table 4). In the MI+ALA+FA group we observed a significantly higher clinical pregnancy rate (CPR) (45% vs. 15%, respectively, *p* < 0.05), a higher ongoing pregnancy rate (OPR; 35% vs. 0%), and a higher live birth rate (LBR; 35% vs. 0).

## 4. Discussion

Obesity has been clearly observed to affect spontaneous female fertility, mainly inducing perturbations of the hypothalamus-pituitary-ovarian axis and producing menstrual dysfunction and anovulation [29]. Furthermore, obese patients were also reported to have poorer IVF outcomes in comparison with women who had a normal weight: higher need of gonadotropins during ovarian stimulation, increased cycle cancellation rate, lower oocyte yield, lower implantation rate, and increased miscarriage rate [30]. In obese women, the adipose tissue releases a number of bioactive molecules, namely adipokines, that variably interact with multiple molecular pathways of insulin resistance, inflammation, hypertension, cardiovascular risk, coagulation, oocyte differentiation, and maturation [31]. Indeed, obesity increases oxidative stress and inflammatory markers in follicular fluid and alters cumulus cell gene expression, affecting mitochondrial function in the oocyte [32,33]. In addition, the altered endocrine status has been reported to display its detrimental effect via interference in the follicular milieu, oocyte maturation, embryo development, and endometrial receptivity [34,35,36,37,38]. 

Myo-Inositol (MI) was demonstrated to exert beneficial effects on overweight/obese women with polycystic ovary syndrome (PCOS), being able to increase mature oocytes yield, oocyte nuclear and cytoplasmic maturation, and finally embryo development [11,39], probably due to its capacity to provide metabolites able to mediate insulin action [40]. However, MI effects on pregnancy and live birth rates after IVF are still a matter of debate [41]. The last Cochrane review, including 13 trials with 1472 subfertile women with PCOS who were receiving MI prior to IVF (11 trials), or during ovulation induction (2 trials), revealed that MI was able to improve clinical pregnancy rate, live birth rate, and miscarriage rate, although with low-quality evidence [42]. Of note, the supplementation of D-chiro-inositol (DCI) with other antioxidant molecules, such as Alpha-Lipoic acid (ALA), led to a lower exogenous gonadotropin need, shorter stimulation, and a higher proportion of mature oocytes and fertilized oocytes in overweight/obese PCOS patients [43]. This evidence prompted us to evaluate the effects of a combination of MI, Alpha-Lipoic acid (ALA), and Folic acid (FA) overweight/obese women not affected by PCOS, who represent a less studied subgroup of IVF patients. We aimed at focusing on oocyte morphological characteristics and embryo in vitro development, who were assessed integrating the conventional morphology with more sophisticated technologies. Polarized light microscopy (PLM), in fact, allows the non-invasive study of anisotropic structures of the oocyte (inner layer of the ZP and MS) that are not visualized by bright field microscopy. The birefringent signals generated by well-organized structures may be measured as light retardance by a specific computerized image-analysis system [44]. Data confirm that PLM may be a useful tool to assess cytoplasmic maturation in order identify oocytes with the best potential for fertilization [45,46], embryo growth [45,47], blastocyst formation [19,48], and implantation [18,49]. 

The results of the present study showed a significantly higher ZP birefringence after MI + ALA + FA intake, suggesting for the first time that the nutraceutical combination may add a beneficial effect on ZP molecular organization during follicular maturation. This hypothesis is supported by our previous observation that ZP structure is physiologically remodeled during oocyte maturation [2], and also by the evidence that MI administration is associated with a higher number of retrieved mature oocytes in PCOS patients [11,50]. Furthermore, it has been observed that ZP birefringence significantly decreases in oocytes from women of advanced reproductive age [51] and, in general, in oocytes with poorer developmental competence [19], originating embryos with lower implantation chance [52]. We also observed a significantly shorter MS axis in the MI + ALA + FA group. Meiotic spindle detection and morphology by PLM during ICSI has been claimed to predict fertilization [53], embryo development [19], and conception [18]. Our previous observations suggest that a MS of larger size is more frequently observed among women of advanced reproductive age and lower ovarian sensitivity to exogenous FSH, suggesting that the increased MS size could represent a marker of oocyte ageing, associated to maturation defects, causing fertilization failure or meiotic errors. Interestingly enough, we also reported that an increased MS size was associated with early embryo development arrest, before reaching the blastocyst stage [3]. Furthermore, the impact of female age on oocyte birefringent structures has been previously described and, in general, considered to be quite limited [3,51,54,55]. In the present study, comparable maturation, fertilization, and cleavage rates were observed in the two groups, suggesting that in the analysed population the woman’s age may have exerted only a limited effect (if any) on oocyte quality and that the significant differences observed in the birefringent structures were likely to be related mainly to factors other than age.

These results are indirectly confirmed by the present observations, showing a significantly higher embryo morphological score and a larger proportion of top-quality embryos at the cleavage stage in the MI + ALA + FA group compared to the FA-only group. 

In the present study, embryo development was assessed using the TLS Geri plus^®^ [20], providing a continuous surveillance of embryo growth, while maintaining stable culture conditions. TLS was claimed to have the potential to improve embryo selection capability and, in turn, IVF outcome [56]; indeed, some studies reported higher pregnancy rates using this technology [57], whereas others did not confirm any advantage vs. standard morphological embryo selection protocols [58,59,60]. Overall, we observed similar embryo morphokinetic parameters between the two study groups, but a significantly higher proportion of embryos displaying an optimal cleavage range in the very early stages of embryo development (t2 and t3) was observed in the MI + ALA + FA group compared to the FA-only group, revealing a potential, although limited, positive impact on embryo kinetics. Interestingly enough, it was previously observed that embryo morphokinetics at the cleavage stage are significantly slower in obese compared with normal-weight women [61], and that embryo development from hyperandrogenic PCOS women is significantly delayed at early stages compared with embryos from non-PCOS, regularly cycling women [62]. Taken together, these data reveal that causative factor for subfertility in overweight/obese or PCOS women may be related, at least in part, to the developmental timing of pre-implantation embryos. However, the exact mechanisms by which maternal overweight and obesity determine slower and potentially poorer embryo development are still unknown, implying that the current data available in literature on overweight/obese patients should be considered with caution. We can only speculate that molecular mechanisms affecting genomic instability may be involved. 

Finally, we accidentally observed a higher implantation rate and cumulative ongoing pregnancy rate in the MI + ALA + FA group compared to the FA-only group. There is abundant scientific evidence that obesity has a detrimental impact on pregnancy chances, affecting both implantation rate and pregnancy loss [63]. Bellver et al. [64] revealed differences in endometrial gene expression during the implantation window in obese patients, especially for those with PCOS. Myo-Inositol has been observed to improve metabolism in women with PCOS and we can hypothesize that its effect may also involve endometrial function in non-PCOS overweight/obese women [65]. At present, the mechanism through which it exerts an action on the endometrium is poorly understood, but we can speculate that this may be mediated, at least in part, by targeting molecules associated to the insulin pathway. In the present study, we observed a significantly higher endometrial thickness in the MI + ALA + FA group, suggesting a more suitable microenvironment for embryo implantation. This hypothesis is further confirmed by the evidence that Myo-Inositol and Alpha-Lipoic acid administration may significantly reduce the endometrial inflammasome, defined as the endometrial pro-inflammatory cytokine system, expression and activation in patients with recurrent miscarriage [66]. Unfortunately, our study was powered to assess oocyte and embryo development characteristics based on previously published power analysis [2,20], and, as a consequence, is highly underpowered to discuss in a meaningful way any clinical variation of IVF outcome. The occasional observation of better outcomes in the MI + ALA + FA group should only be intended as a preliminary finding, encouraging the design of further studies with an adequate number of observations to investigate the effects of MI + ALA + FA on clinical IVF results. In addition, we recognize that our observations may be of interest only for those laboratories evaluating oocyte morphology using PLM and performing morphokinetic analysis of embryo development using the same time-lapse system.

## 5. Conclusions

In this prospective randomized pilot study, we report for the first time that the combination of Myo-Inositol, Alpha-Lipoic acid and Folic acid may have a positive effect on oocyte morphological features and embryo morphokinetics in non-PCOS, overweight/obese women undergoing IVF. Our observations encourage testing of the effects of this nutraceutical combination on the clinical outcome of IVF, designing adequately powered, further randomized trials. Furthermore, it would also be interesting to test the effect of the combination of the three compounds on the quality of oocytes and embryos in non-obese, infertile women. 

## Figures and Tables

**Table 1:** Patients’ clinical characteristics.

	MI + ALA + FA (*n* = 20)	FA-Only (*n* = 20)	*p*
Age (years)	35 ± 5.5	37.2 ± 3.3	ns
BMI (kg/m^2^)	29.9 ± 2	29.6 ± 2.3	ns
AMH (ng/mL)	2.9 ± 2.6	2.8 ± 2.6	ns
AFC (*n*)	15.9 ± 8.9	14.3 ± 7.4	ns
Total exogenous FSH (IU)	3090 ± 1000	3053 ± 890.3	ns
OSI (*n*)	2.8 ± 2	3.3 ± 2.3	ns
Peak E2 (pg/mL)	1735 ± 689	2286 ± 1147	ns
Endometrial thickness (mm)	11.4 ± 2.2	9.9 ± 1.8	<0.05
Retrieved oocytes (*n*)	7.8 ± 4	8.5 ± 4.3	ns
Mature (MII) oocytes (*n*)	6.6 ± 3.3	7.3 ± 4	ns
Maturation rate (%)	84.3 ± 17.2	87.3 ± 16.2	ns
Fertilized oocytes (*n*)	4.3 ± 2.5	4.7 ± 3.1	ns
Fertilization rate (%)	69.9 ± 25	64.6 ± 20.1	ns
Cleaved embryos (*n*)	4.2 ± 2.4	4.6 ± 3.2	ns
Cleavage rate (%)	98.5 ± 4.8	97.4 ± 8.4	ns
Mean embryo score	7.4 ± 2.3	6.7 ± 2.1	<0.01
Top quality embryos (*n*)	1.9 ± 1.5	1.2 ± 1.8	ns
Top quality embryos (%)	45.2 (38/84)	26.1 (24/92)	<0.01

BMI = body mass index; AFC = antral follicle count; E2 = estradiol; OSI = ovarian sensitivity index (number of retrieved oocytes × 1000/total FSH dose). MI + ALA + FA group = patients assuming Myo-Inositol + Alpha-Lipoic acid + Folic acid; FA-only group = patients assuming Folic acid alone. Data are shown as mean ± SD or as absolute values and percentage; ns = not significant.

**Table 2:** Morphological features of metaphase II (MII) oocytes measured by Polarized Light Microscopy (PLM).

	MI+ALA+FA (*n* = 155)	FA-Only (*n* = 169)	*p*
IL-retardance (nm)	2.1 ± 0.6	1.9 ± 0.5	<0.001
IL-area (µm^2^)	2895 ± 574	2574 ± 491	<0.0001
IL-thickness (µm)	5.3 ± 1.4	4.5 ± 1.2	<0.05
MS-retardance (nm)	1.6 ± 0.5	1.6 ± 0.4	ns
MS-area (µm^2^)	85.9 ± 23.4	89.3 ± 21.9	ns
MS-axis (µm)	11.8 ± 1.8	12.3 ± 2.3	<0.05

MI + ALA + FA group = patients assuming Myo-Inositol + Alpha-Lipoic acid + Folic acid; FA-only group = patients assuming Folic acid alone. IL = inner layer; MS = meiotic spindle. Values are expressed as mean ± standard deviation; ns = not significant.

**Table 3:** Morphokinetic parameters recorded by TLS GERI plus^®^.

	MI+ALA+FA (*n* = 84)	FA-Only (*n* = 92)	*p*
tPNa (h)	7.9 ± 1.8	7.5 ± 1.7	ns
tPNf (h)	23.3 ± 3.9	22.7 ± 3.6	ns
t2 (h)	26.6 ± 3.8	25.7 ± 3.7	ns
t3 (h)	36.2 ± 3.7	35.8 ± 4.8	ns
t4 (h)	38.3 ± 5.4	38.5 ± 6.6	ns
t8 (h)	61.6 ± 12.4	58.8 ± 11.1	ns
tSB (h)	102.5 ± 8.2	98.9 ± 10.3	ns
tB (h)	115.6 ± 8.3	117.2 ± 10.2	ns
tPNf–tPNa (h)	15.3 ± 3.5	15.2 ± 3.9	ns
t2-tPNf (h)	3.4 ± 1.6	3 ± 0.7	ns
t3-t2 (h)	9.6 ± 3.9	10.4 ± 3.6	ns
t4-t3 (h)	2.7 ± 5.1	2.8 ± 5.9	ns
t4-t2 (h)	12.2 ± 4.8	13.1 ± 5.9	ns
t8-t4 (h)	22.8 ± 13.3	20.5 ± 8.9	ns
t2 (24.3–27.9 h)	51.1 (43/84)	33.6 (31/92)	<0.05
t3 (35.4–40.3 h)	47.6 (40/84)	25 (23/92)	<0.01
t4 (36.4–41.6 h)	40.4 (34/84)	28.2 (26/92)	ns
t3–t2 (≤ 11.9 h)	75 (63/84)	70.6 (65/92)	ns
t4–t3 (≤ 0.76 h)	38 (32/84)	47.8 (44/92)	ns

Times and time intervals are shown for embryos of the MI + ALA + FA group (patients assuming Myo-Inositol + Alpha-Lipoic acid + Folic acid) and of the FA-only group (patients assuming Folic acid alone). Data are shown as mean ± standard deviation or as absolute values and percentage; ns = not significant.

**Table 4:** Clinical outcome of intracytoplasmic sperm injection (ICSI) according to the assumption of Myo-Inositol + Alpha-Lipoic acid + Folic acid (MI + ALA + FA group) or Folic acid alone (FA-only group).

	MI+ALA+FA (*n* = 20)	FA-Only (*n* = 20)	*p*
Transferred embryos (n)	30	28	
Implantation rate (%)	33.3 (10/30)	10.7 (3/28)	<0.05
Clinical pregnancy rate (CPR) (%)	45 (9/20)	15 (3/20)	<0.05
Ongoing pregnancy rate (OPR) (%)	35 (7/20)	0 (0/20)	-
Live birth rate (cLBR) (%)	35 (7/20)	0 (0/20)	-

Data are expressed as absolute values and percentages.
